# Behavioural phenotypes over the lifetime of a holometabolous insect

**DOI:** 10.1186/1742-9994-12-S1-S8

**Published:** 2015-08-24

**Authors:** Thorben Müller, Caroline Müller

**Affiliations:** 1 Department of Chemical Ecology, Bielefeld University, Universitätsstr. 25, 33615 Bielefeld, Germany

**Keywords:** *Phaedon cochleariae*, Holometabolous insect, Behavioural dimensions, Individuality, Activity, Boldness, Lifetime, Metamorphosis, Ontogeny

## Abstract

Introduction: Behavioural traits can differ considerably between individuals, and such differences were found to be consistent over the lifetime of an organism in several species. Whether behavioural traits of holometabolous insects, which undergo a metamorphosis, are consistent across ontogeny is virtually unexplored. We investigated several behavioural parameters at five different time points in the lifetime of the holometabolous mustard leaf beetle *Phaedon cochleariae* (Coleoptera: Chrysomelidae), two times in the larval (second and third larval stage) and three times in the adult stage. We investigated 1) the stability of the behavioural phenotype (population level), 2) whether individuals rank consistently across behavioural traits and over their lifetime (individual level), and 3) in how far behavioural traits are correlated with the developmental time of the individuals.

Results: We identified two behavioural dimensions in every life stage of *P. cochleariae*, activity and boldness (population level). Larvae and young adults ranked consistently across the investigated behavioural traits, whereas consistency over time was only found in adults but not between larvae and adults (individual level). Compared to adult beetles, larvae were less active. Moreover, younger larvae were bolder than all subsequent life stages. Over the adult lifetime of the beetles, males were less active than females. Furthermore, the activity of second instar larvae was significantly negatively correlated with the development time.

Conclusions: Our study highlights that, although there is no individual consistency over the larval and the adult life stage, the behavioural clustering shows similar patterns at all tested life stages of a holometabolous insect. Nevertheless, age- and sex-specific differences in behavioural traits occur which may be explained by different challenges an individual faces at each life stage. These differences are presumably related to the tremendous changes in life-history traits from larvae to adults and/or to a niche shift after metamorphosis as well as to different needs of both sexes, respectively. A faster development of more active compared to less active second instar larvae is in line with the pace-of-life syndrome. Overall, this study demonstrates a pronounced individuality in behavioural phenotypes and presumably adaptive changes related to life stage and sex.

## Introduction

An organism in its environment is influenced by several abiotic and biotic factors, which may affect various life-history traits during the whole ontogeny, including growth parameters and behaviour [1-3]. Within animal populations, pronounced individual differences in behaviour can occur, often described as ‘personality’ [4]. Personality is defined as behaviour which is consistent within individuals over time and across different contexts [4-6]. Consistent individual differences in behaviour can influence the fitness of each individual and, on a larger scale, the distribution as well as the ability of a species to adapt to environmental changes [7].

While personality has been found in various vertebrate and invertebrate animals [8], the stability of these behavioural phenotypes over the entire ontogeny has received much less attention [7,9]. However, behavioural phenotypes may emerge during ontogeny [6,9] and may change or need to be adapted at critical stages of the lifetime, for example, during adolescence, when animals reach sexual maturity [10-13] or when the season changes [14]. Animals exhibit an enormous array of life-histories which offers the opportunity to study stabilities or changes in behavioural traits over the entire ontogeny of an organism. For such studies, invertebrates are convenient model organisms due to their usually shorter life span compared to vertebrates [15,16]. Particularly in holometabolous insects, the metamorphosis is a switch point, at which the organism is thoroughly reorganised. Nevertheless, surprisingly little is known yet about within- and between-individual variation in animal behavioural phenotypes of invertebrates [16]. With regard to insects, mainly hemimetabolous insects were studied [17-19], whose life-history trajectories are, however, more stable than that of holometabolous insects (but see [20]).

Possible behavioural changes over the lifetime could be influenced by changes in hormonal profiles, physiology, and morphology [21-23], especially in organisms which undergo a metamorphosis [23,24]. In most insect species, the larvae or juvenile stages are much less mobile than adults and may be subject to different predators, with juveniles often suffering higher predation risks [25]. Furthermore, larvae mainly search for food, whereas adults also search for mating partners and females need to find oviposition sites. Such changes in the ecological needs or niche shifts in combination with growth and development of an organism may affect an animal's optimal behaviour [26]. As a consequence, the selection pressure on boldness and activity can vary over a lifetime [19,25,27]. For example, juveniles of hemimetabolous firebugs (*Pyrrhocoris apterus*, Heteroptera: Pyrrhochoridae) and crickets (*Gryllus integer*, Orthoptera: Gryllidae) are bolder and firebugs are more active and explorative than adult individuals [19,28]. In the fishing spider *Dolomedes triton* (Araneae: Pisauridae), no boldness syndrome is found in juveniles but after the adult moult it becomes evident [29]. In contrast, in the damselfly *Lestes congener *(Odonata: Lestidae) activity and boldness in the adult stage are correlated with the behavioural phenotype of the larvae, even though larvae live aquatic, whereas adults are terrestrial [30]. This result shows that also in insects which undergo a metamorphosis the behavioural phenotype can be carried over from the larval to the adult stage [30].

Differences in behavioural traits between individuals might also be explained by life-history trade-offs, for example, between growth and mortality [31], as considered in the pace-of-life syndrome (POLS) [32]. The POLS integrates behavioural traits with differences in growth rates at different life stages and with physiological differences [32,33]. For example, a fast pace-of-life is predicted to combine high activity, boldness, and aggressiveness with high metabolic rates and a low immune responsiveness [32].

In the present study, the stability of the behavioural phenotype over ontogeny was tested in the holometabolous mustard leaf beetle* Phaedon cochleariae* (F.) (Coleoptera: Chrysomelidae), considering the individual and the population level. Recently, it has been demonstrated that the behaviour of adult mustard leaf beetles is consistent across contexts and over a period of about three weeks [3]. To investigate how the behavioural phenotype develops over nearly the entire ontogeny, here we performed a set of behavioural assays two times in the larval stages and three times over an extended period of the adult life. We predicted that larvae would be bolder and more active than adults, as found in some hemimetabolous insect species [19,28]. Furthermore, we expected that males and females may differ in behaviours due to different requirements. Finally, we investigated whether there is a relationship between behavioural traits and the developmental time, which would point to the POLS.

## Results

### Development time

Of 65 freshly hatched *P. cochleariae *larvae, which were kept individually in Petri dishes on cabbage, 52 successfully developed into adults. The development time from freshly hatched larvae to adult hatching ranged from 19-23 days (20.68 ± 0.85 days, mean + SD) and did not differ between males and females (Mann Whitney-*U* test, U = 306.5, *p* = 0.712, *N*_females_ = 21, *N*_males_ = 31).

### Analysis of behavioural traits

**Figure 1:**
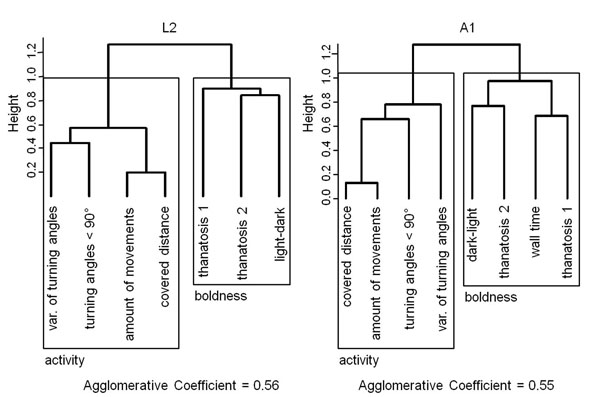
Agglomerative cluster analysis of behavioural traits measured left) in L2 larvae and right) in A1 adults of *Phaedon cochleariae* (L2,* N* = 35; A1,* N* = 48). The height is based on a Spearman rank correlation matrix among behavioural traits. The number of marked behavioural dimensions (activity and boldness) was identified by a Silhouette plot (not shown). Var. - variance.

Behavioural traits were measured five times throughout the lifetime, testing up to eight traits in up to four different contexts. Six behavioural traits were determined for both larvae and adults. The traits *covered distance, amount of movements, turning angles < 90°* and *variance of turning angles* were received from video analyses of the locomotion of individuals in Petri dishes within 30 min (larvae) or 1 h (adults), respectively. The duration of thanatosis (tonic immobility; *thanatosis 1*) and the subsequent activity time (*thanatosis 2*) were measured after imitating a predator attack. Furthermore, for larvae the latency of reaching a safe refuge in an unprotected environment (*light-dark* test) was measured. Alternatively, for adults the latency of leaving a safe refuge (hiding context, *dark-light* test) and of reaching a safe wall in an unprotected environment context (*wall time* test) were determined. Applying an agglomerative cluster analysis on the measured behaviours combined with a Silhouette plot to determine the likely number of behavioural groups for each life stage separately, two dimensions of behavioural traits could be identified for L2 and L3 larvae as well as young adults (10-17 d old, A1) at the population level (Fig. 1). In the larval stage L2 as well as in the A1 adults, the behavioural traits *covered distance, amount of movements, turning angles < 90°*, and *variance of turning angles* were clustered together and defined as the activity dimension (Fig. 1). Furthermore, for the larvae the traits *light-dark, thanatosis 1*, and* thanatosis 2 *were clustered together and formed a second dimension termed boldness (Fig. 1, left). This dimension could be considered as equivalent to the traits *dark-light, wall time, thanatosis 1*, and* thanatosis 2* which clustered in A1 adults (Fig. 1, right). The behavioural traits for L3 larvae clustered in the same dimensions as traits of L2 larvae (L3, agglomerative coefficient = 0.55). Thus, at the population level, the behavioural clustering showed similar patterns. The behavioural traits of older adults (24-31 d old, A2; 38-45 d old, A3) clustered almost identically as in A1 adults except that the trait *turning angles < 90° *was grouped in the boldness dimension (A2, agglomerative coefficient = 0.53; A3, agglomerative coefficient = 0.52). The agglomerative coefficients were similar or higher to those found in comparable studies and indicate a reasonable clustering [3,17].

### Consistency across behavioural traits and contexts

Comparing ranks of individual measurements with Kendall's W coefficient of concordance in the L2 and L3 larvae as well as in the A1 adults at the individual level, there was a consistency over all seven (larvae) or eight (adults) behavioural traits which were derived from three (larvae) or four (adults) contexts (Table 1). Likewise, consistency across the behavioural traits of the activity dimension (tested in one context) was found, but not among the behavioural traits of the boldness dimension (two contexts for larvae and three for adults) when these traits were tested separately. In contrast, in the A2 and A3 adults no consistency across the behavioural traits was found, neither between the eight tested behavioural traits nor among traits within the activity and boldness dimension alone (Table 1).

**Table 1:** Consistency of individual ranks over all tested behavioural traits measured in three contexts for larvae and four for adults of *Phaedon cochleariae* (L2,* N* = 35; L3,* N* = 51; A1,* N* = 48; A2,* N* = 45; A3,* N* = 41). Consistency was tested using Kendall's coefficient of concordance *W. P*-values remaining significant after correction for false discovery rate within a lifestage are indicated in bold; the uncorrected *P*-values are shown.

	Test across behaviours	*W*	*p*-value
L2	All 7 behavioural traits	0.368	**< 0.001**
	4 traits for activity	0.822	**< 0.001**
	3 traits for boldness	0.328	0.496
L3	All 7 behavioural traits	0.296	**< 0.001**
	4 traits for activity	0.713	**< 0.001**
	3 traits for boldness	0.213	0.978
A1	All 8 behavioural traits	0.189	**0.013**
	4 traits for activity	0.415	**0.003**
	4 traits for boldness	0.288	0.220
A2	All 8 behavioural traits	0.135	0.333
	3 traits for activity	0.311	0.601
	5 traits for boldness	0.222	0.288
A3	All 8 behavioural traits	0.156	0.137
	3 traits for activity	0.223	0.947
	5 traits for boldness	0.233	0.218

### Consistency over time and age-dependent behavioural differences

Composite variables were calculated per life stage by adding the ranks of each individual over all behavioural traits, which clustered in one dimension. The larval behaviour was neither consistent over time between L2 and L3 (i.e., individual behavioural traits as well as composite variables for activity and boldness), nor between L3 and the A1 adults (i.e., individual behavioural traits as well as composite variable activity) (Table 2), tested by Kendall's W coefficients of concordance. In contrast, adult beetles behaved consistently across adulthood from A1 to A3 in all behavioural traits except *dark-light*, *thanatosis 2*, and *wall time* (Table 2).

**Table 2:** Consistency of individual ranks in behavioural traits over time in larvae and adults of *Phaedon cochleariae* (L2-L3,* N* = 31, L3-A1,* N* = 41, A1-A3,* N* = 38). The consistency over time was tested for all possible behavioural traits between the stages using Kendall's coefficient of concordance W. *P*-values remaining significant after correction for false discovery rate within behavioural dimensions are indicated in bold; the uncorrected *P*-values are shown.

	Test for consistency over time	W	*p*-value
L2-L3	**Activity (composite variable)**	0.675	0.096
	*Covered distance*	0.579	0.253
	*Amount of movements*	0.661	0.111
	*Turning angles < 90°*	0.574	0.263
	*Variance of turning angles*	0.633	0.150
	**Boldness (composite variable)**	0.486	0.508
	*Light-Dark*	0.390	0.797
	*Thanatosis 1*	0.664	0.108
	*Thanatosis 2*	0.631	0.153
L3-A1	**Activity (composite variable)**	0.576	0.235
	*Covered distance*	0.573	0.242
	*Amount of movements*	0.561	0.275
	*Turning angles < 90°*	0.597	0.186
	*Variance of turning angles*	0.413	0.776
	*Thanatosis 1*	0.518	0.408
	*Thanatosis 2*	0.553	0.296
A1-A3	**Activity (composite variable)**	0.635	**< 0.001**
	*Covered distance*	0.684	**< 0.001**
	*Amount of movements*	0.792	**< 0.001**
	*Turning angles < 90°*	0.616	**0.001**
	*Variance of turning angles*	0.6	**0.002**
	**Boldness (composite variable)**	0.751	**0.005**
	*Dark-Light*	0.463	0.058
	*Wall Time*	0.405	0.172
	*Thanatosis 1*	0.572	**0.004**
	*Thanatosis 2*	0.46	0.062

**Figure 2:**
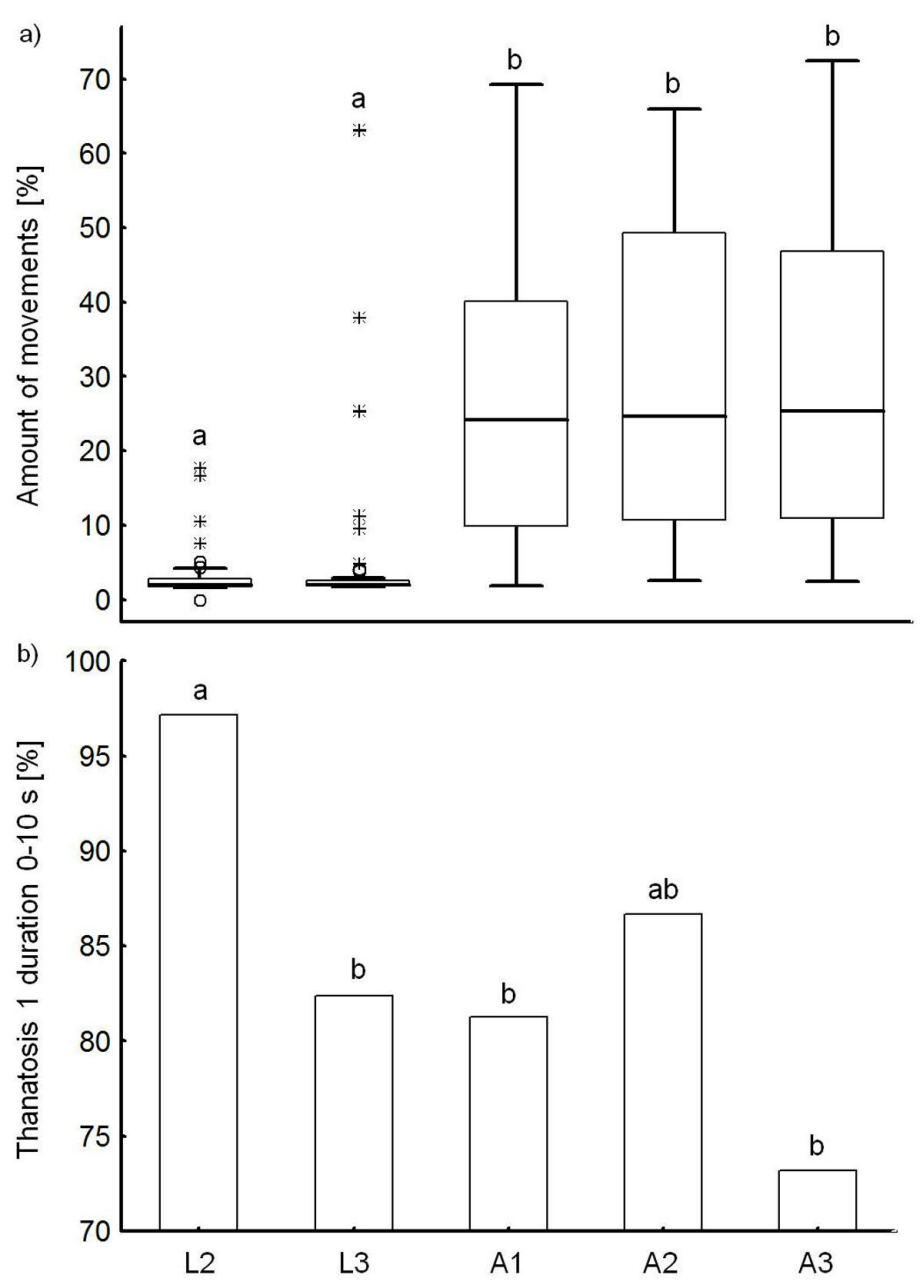
Changes in a) a typical activity trait (*amount of movements*) and b) a typical boldness trait (proportion of individuals showing a *thanatosis 1* duration of ≤ 10 s) over the lifetime of *Phaedon cochleariae * (L2, *N* = 35; L3, *N* = 51; A1, *N* = 48; A2, *N* = 45; A3, *N* = 41). Lower case letters indicate significant differences (*p*< 0.05) between the *amount of movements* (one way repeated measures ANOVA, followed by pairwise multiple comparisons, Holm-Sidak method) and the ratio of individuals with a short *thanatosis 1* duration (Fischer's exact tests, comparing data between larval stages and between larval and adult stages; after correction for false discovery rate only the difference between L2 and A3 remained significant). The box plots show the median (line), the 25th and 75th percentiles, outliers (circles), and extreme values (asterisks).

The *amount of movements*, chosen as typical activity trait, was significantly lower in the larval compared to the adult stages in individuals measured throughout life (one way repeated measures ANOVA, life stage* F*_4,153_= 33.619, *p* < 0.001, followed by pairwise multiple comparisons, Fig. 2a). The proportion of individuals showing a *thanatosis 1 *duration of ≤ 10 s (fast responders) compared to > 10 s (slow responders), chosen as a typical boldness trait, differed significantly between L2 and L3 larvae (Fisher's exact test,* p* = 0.043; *N*_L2_ = 35, *N*_L3_ = 51), L2 and A1 (*p* = 0.039; *N*_A1_ = 48) and L2 and A3 (*p* = 0.004; *N*_A3_ = 41), respectively. After correction for false discovery rate, only the difference between the L2 larvae and the A3 adults remained significant. Most L2 larvae showed only very short * thanatosis 1* durations, whereas latencies increased with age, with A3 adults showing more often durations > 10 s (Fig. 2b).

### Sex-dependent behavioural differences

**Figure 3:**
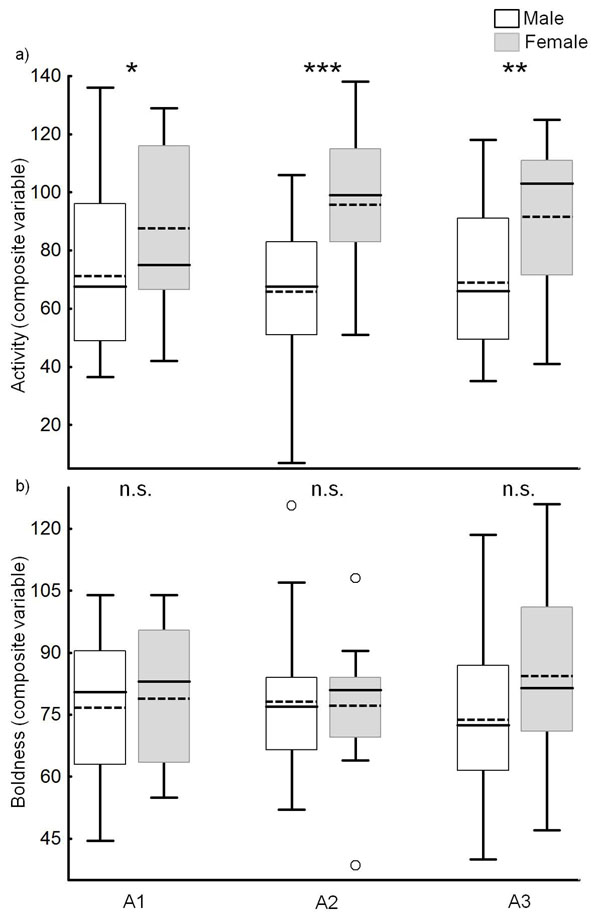
Differences in a) activity and b) boldness between female and male adults of * Phaedon cochleariae* (males, *N* = 23; females, *N* = 15). The composite variable activity included the behavioural traits *covered distance*, *amount of movements*, *turning angles < 90°*, and *variance of turning angles*. The composite variable boldness included the behavioural traits *dark light*, *wall time*, *thanatosis 1* and *2*. The asterisks indicate significant differences (* *p*< 0.05, ** *p*< 0.01, *** *p*< 0.001) between the activity of males and females(two way repeated measures ANOVA, followed by pairwise multiple comparisons, Holm-Sidak method; there was no significant difference between beetles of different age); n.s. - not significant. The box plots show the median (line), mean (dotted line), the 25th and 75th percentiles, and outliers (circles).

Adult females showed a significantly higher activity (composite variable) than males throughout adulthood, but the activity did not differ between adults of different age (two way repeated measures ANOVA; sex,* F*_1,36_ = 13.355, *p*< 0.001; age,* F*_2,72_ = 0.045, *p* = 0.956; sex x age,* F*_2,72_= 1.020, *p* = 0.366; followed by pairwise multiple comparisons; Fig. 3a). In contrast, the composite variable boldness did neither differ between sexes nor between beetles of different age (two way repeated measures ANOVA; sex,* F*_1,36_ = 0.767, *p* = 0.387; age,* F*_2,72_= 0.062, *p* = 0.940; sex x age,* F*_2,72_ = 1.398, *p* = 0.254; Fig. 3b).

### Correlation between development time and behavioural dimensions

**Figure 4:**
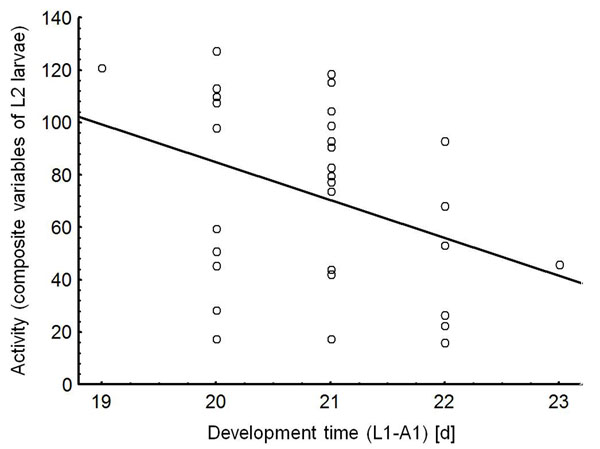
Correlation between the development time (from first larval stage until adult emergence) and activity. The composite variable activity included the behavioural traits* covered distance*, *amount of movements*,* turning angles < 90°*, and *variance of turning angles *of L2 larvae of *Phaedon cochleariae *(*N* = 31). Regression line based on a Spearman rank correlation, *r* = -0.358, *p* = 0.048.

The development time (from hatching of larvae until adult emergence) and the composite variable activity of L2 larvae were significantly correlated (Spearman rank correlation, *r* = -0.358, *p* = 0.048, *N* = 31) (Fig. 4). No correlations were found between the developmental time and activity of L3 larvae (*r* = 0.018, *p* = 0.903, *N* = 46) and between the developmental time and boldness of L2 (*r* = -0.118, *p* = 0.525) and L3 larvae (*r* = -0.260, *p* = 0.080), respectively.

## Discussion

Across the population level, the behavioural dimensions activity and boldness were found in all investigated larval instars and adult life stages of *P. cochleariae*. At the individual level, the rank order of each larva and young adult was highly consistent across behavioural traits measured in different contexts, but neither consistent over time among the larval stages nor between the larval and the adult life stage. Instead, we detected significant differences in typical activity and boldness traits between the stages. The data show that behaviour can be shaped during ontogeny. Specific events during the sensitive larval stage may influence the further behavioural development, resulting in a more tightened behavioural phenotype in the adult stage (individual consistency over time of adult leaf beetle behaviour). Further studies are needed to investigate whether mainly larval and/or adult experiences shape adult behaviour. The results of the present study are in line with other studies which demonstrated that insects show individual behavioural phenotypes that are characterised by stage-dependent and age-specific differences in several behavioural traits [26,28,30]. However, our results highlight to our knowledge for the first time how the behavioural phenotype, characterised by up to eight behavioural traits organised in two behavioural dimensions, develops nearly over the complete lifetime in a holometabolous insect.

### Stable behavioural structure through out the lifetime

Throughout the ontogeny of *P.cochleariae*, two behavioural dimensions, activity and boldness, were concordantly found at the population level. In two earlier studies on adult behavioural phenotypes of this beetle species and another chrysomelid, the traits, which clustered in the activity dimension in the present study, were split in two behavioural dimensions, with the activity dimension being characterised by the *covered distance* and the* amount of movements*, whereas an additional non-targeted explorativeness cluster comprised the traits *turning angles < 90° *and *variance of turning angles *[3,34]. The different assignment of traits to behavioural dimensions in the earlier studies [3,34]compared to the present study may be explained by minor differences in some behavioural tests or slight differences in food quality offered to the insects. For example, *thanatosis 2 *was exclusively measured in the present study, whereas the movement in the inner area of a Petri dish was only recorded in the earlier studies on chrysomelid beetles [3,34]. Furthermore, beetles were fed either young or old leaves in the earlier studies [3,34], whereas food was provided in alternating order in the present study.

A stable correlation structure between various behavioural traits has also been found earlier for some hemimetabolous insects [28,30]. Our results show that even for insects which are holometabolous the behavioural phenotype can be stable throughout the lifetime. Such persistence in behavioural structure is not necessarily found throughout life in invertebrate species (e.g., not in spiders, see introduction [29]). More studies are needed that investigate the stability of behavioural phenotypes of various invertebrate species to determine whether pattern are taxon-specific or differ in general between animals with contrasting life-histories such as spiders, hemimetabolous, and holometabolous insects.

### Age- and sex-dependent behavioural differences

Although the correlation structure of the two behavioural dimensions remained stable over the lifetime of *P. cochleariae*, the rank order in different behavioural traits was not consistent over time at the individual level, neither among the two tested larval stages nor between the larvae and the adults. In particular, age-dependent differences were found in activity (represented by *amount of movements*) as well as in boldness (represented by *thanatosis 1*). Whereas adult beetles were generally more active than larvae, L2 larvae were boldest. Such differences between life stages could be explained either by the stage-specific life-history (differences in anatomy, morphology, and physiology), by special challenges or needs at each specific life stage, and/or by ecological niche shifts between life stages. Thus, different levels of the same behaviour could be advantageous [35], leading to distinct responses of juvenile instars and adults in the same situation [28]. Since juveniles should mainly be adapted to growth and survival and the last larval instar additionally needs to find a site for pupation, whereas adults should maximise their reproductive output [36,37], from a life-history perspective they are expected to respond differently.

Larvae of *P. cochleariae *may be less active than adults simply because they are less mobile. In contrast, adults should be more active since they need to find mating partners and oviposition sites, and since they may disperse. Although adult beetles have elytra, we never observed *P. cochleariae *flying. Nevertheless, in this experiment beetles moved distances with up to 13.5 m/h (personal observation). The bolder behaviour of L2 larvae in comparison to adult beetles may be explained by the asset protection principle [35,38]. Adult beetles have more to lose if they become victims of predators because they are in the reproductive stage [35,39]. Furthermore, in the L2 stage, larvae are much lighter than L3 larvae and adult beetles of *P. cochleariae*. As a consequence, it is most important for young larval instars to gain weight and they cannot afford to show long thanatosis after a predator attack, since they need to forage intensely. Heavier L3 larvae and adult beetles are probably able to cope with longer periods of time without feeding. Moreover, thanatosis may be inefficient in young larvae, since they probably face a larger range of different predators than adults. Similar age- and stage-dependent differences in several characteristic behavioural traits have been found in some hemimetabolous insect species [19,26,28], even though hemimetabolous insect larvae change their overall phenotype much less than holometabolous insects.

Within the adult stage of *P. cochleariae*, a high rank order consistency was found across time in almost all investigated behavioural variables. Thus, results of an earlier study carried out with adult beetles of this species over a period of about three weeks [3] could be confirmed for an even longer period of almost five weeks in the present study, representing almost the entire adult life span. In conclusion, selection might uncouple the behavioural phenotypes over the lifetime if environmental conditions and functional needs of juveniles differ from those of adults [7], but within the adult phase they may be stable, if the environment does not change.

However, sex-dependent differences were found in adult *P. cochleariae*, with females being more active (composite variable) than males. The activity differences between the sexes might be due to different challenges or functional needs of females compared to males. Females are generally heavier than males and hence might have higher food requirements. Additionally, females need to find appropriate oviposition sites and accordingly may benefit from a higher searching activity effort. In contrast, we could not detect any sex-dependent differences in boldness of mustard leaf beetles. So far, most behavioural differences between the sexes of insects have been found for boldness traits [17,19]. For example, females of the long-winged morph of firebugs are bolder than males [17]. Boldness behaviour of female field crickets is repeatable over the lifetime, whereas males become less bold with maturation, which might be explained by the risky courtship songs of the males which could attract predators [19].

### Correlations between development time and behaviour in the larval stage

The activity (composite variable) of L2 larvae was significantly correlated with the developmental time from the first larval stage until hatching of the adult beetles. In other words, larvae which developed faster were more active. This result supports the POLS hypothesis, which states that a shorter development time and consequently a faster growth rate is positively correlated with activity and hence potentially a higher dispersal ability [32]. The fact that only development time and activity of L2, but not that of L3 larvae, were correlated might be explained by stage-specific features or challenges. At the end of the third instar, larvae become quite inactive and do not feed any more before pupation. In contrast, since small larvae are most vulnerable to different predators, they should develop quickly to outgrow predation risk from smaller predators. Thus, high activity during the L2 stage might be crucial to increase the probability to survive this stage. Additionally, a more active behaviour may result in higher energy gains if more food is foraged and consequently may result in a shorter development time [40].

Interestingly, in *P. cochleariae* activity and boldness are not necessarily related, since we did not find a correlation between boldness and the developmental time. Environmental conditions can even lead to opposite responses in activity and boldness in this beetle [3]. Until now, only few empirical studies exist about possible correlations between different behavioural dimensions and life-history parameters in invertebrates. Faster growing crickets mature earlier and invest less in immune defence in comparison to slower growing individuals [26]. Red morphs of the pea aphid (*Acyrthosiphon pisum*) show a strong trade-off between early reproduction and their life span which, moreover, correlates with their boldness behaviour [41]. In contrast to invertebrates, in many vertebrates such relationships, mostly between boldness and reproduction or growth, have been well described [42-44].

## Conclusions

In conclusion, we demonstrate that the clustering of behaviours can be stable throughout the lifetime of a holometabolous leaf beetle at the population level. Rank order differences in activity and boldness traits of the individuals could be related to the physical abilities at a certain age, the ecological niche the individual is realising, and challenges it faces. Since we found age-dependent differences in specific behavioural traits, the role of the larval stage for the future behavioural phenotype should be studied in more detail, for example, by using match-mismatch experiments [45]. Our study offers some insights how the behavioural phenotypes of organisms develop over the entire lifetime, but it also reveals the need for further comparative studies with various invertebrates and possibly vertebrates to determine potentially common patterns and to advance the understanding of this aspect of individuality in behavioural biology.

## Materials and methods

### Study organism and rearing conditions

The chrysomelid beetle *P. cochleariae *is a specialist on species of the Brassicaceae family and can reach pest status on some crops [46]. Half of the adults survive for up to 60 days under our laboratory conditions in this experimental set-up. Beetles were collected in different parts of Germany and reared for several generations under constant conditions in a climate cabinet (20°C, L16:D8, 65% r.h.). The beetles were kept in ventilated plastic boxes (20 x 20 x 6.5 cm) and provided with leaves of *Brassica rapa* L. ssp. *pekinensis* var. Michihili (Brassicaceae)* ad libitum*. Food plants were grown from seeds (Kiepenkerl, Bruno Nebelung GmbH, Konken, Germany) in pots (12 cm diameter) filled with composted soil in a greenhouse (L16:D8, 60% r.h.). Leaves of non-flowering, 8-10 week old plants were used as food.

### Experimental set-up

To investigate the development time and the behavioural phenotype of *P. cochleariae*, 65 neonate larvae were kept individually in Petri dishes (5.5 cm diameter) covered with plastic cups (4.5 cm diameter, 5 cm height). Cabbage leaf disks (2.5 cm diameter) supported with moistened sponge rubber to prevent desiccation were offered. The insects received young and mature cabbage leaves in an alternating order to supply them with food of mixed quality. For larvae, food was switched every day, for adult beetles every other day. The development time from the first larval stage until hatching of the adult beetles was recorded. After metamorphosis, adult beetles were sexed and kept in pairs of one female and one male.

The behavioural traits of individuals were investigated in a test series five times, two times in the larval stages [second (L2) and third larval instar (L3)] and three times during adulthood [between day 10-17 (A1), day 24-31 (A2) and day 38-45 (A3) of adult life]. Since the L2 larval stage only lasts for three to four days, 35 L2 larvae could be tested for their behaviour within this short time period. In the subsequent live stages, behavioural tests were performed with 41-51 individuals. For activity measurements of larvae and behavioural tests of adults, we used a similar test series as described earlier [3]. To test behavioural traits in a locomotion context, the movements of individual larvae in a Petri dish (9 cm diameter) were observed for 30 min and that of adults for 60 min with a camera (Life Cam VX-2000, Microsoft, Seattle, WA, USA). The observation time differed since larvae cannot starve as long as adults. Using the motion detection software Cam Alert (2.9.23, Max Christian Pohle) and the programs Power batch (Power batch 6.1.2.2, UniDream Marketing Technologies Inc) and Virtual Dub 1.9.11 (Avery Lee) a video sequence was created. Applying the software Bug Tracer Program (version C, Robert Winkler, based on a MATLAB motion detection script of Lokesh Peddireddi) the traits *covered distance* [cm], relative* amount of movements* (percentage of time an individual is moving), *number of turning angles* < 90°, and *variance of turning angles* were revealed. After motion detection, individuals had a 30 min resting period for feeding. Afterwards, three additional behavioural traits were measured in larvae and four in adults, using tests which are appropriate to the respective life stage [30]. All following tests were ended after 300 s irrespective of whether the individual had finished the tasks or not. In a light-dark test, only performed with larvae, the latency (*light-dark*) needed to reach a safe refuge within a Petri dish (9 cm diameter) was measured. The safe refuge consisted of a round black cardboard disk (2 cm diameter), which was accessible from two sites 1 cm off the ground and in a distance of 1 cm from the edge of the Petri dish. The larva was inserted at the opposite site from the refuge in the Petri dish. In contrast, adults were tested in a dark-light test. In this test, beetles were put in a brown glass vial (1 cm diameter, 6 cm height) for one minute. Thereafter, the vial was shortly shaken, then placed horizontally and the latency the beetle needed to completely exit the vial was recorded (*dark-light*). The light-dark and dark-light test served to investigate the behaviour in an unprotected environment (larvae) or hiding context (adults), respectively. The dark-light test was followed by a wall time test to investigate the adult behaviour in the context of an unprotected environment. In the wall time test, a beetle was placed in the middle of an arena (17.2 cm diameter) surrounded by a wall (0.8 cm height) and the latency the beetle needed to reach the wall was determined (*wall time*). Finally, a thanatosis test was performed with larvae and adults after a 15 min break to recover from the previous tests. To test the behaviour in a predator response context, the individuals were gently squeezed with spring steel forceps inducing a thanatosis reaction (tonic immobility). The duration of thanatosis reaction (*thanatosis 1*) and the duration until the beetle stopped again after moving (*thanatosis 2*) were recorded. We interpret a longer lasting *thanatosis 2 *duration as a bolder behaviour, since the individual does not seem to be long-term affected by a predator attack under these circumstances.

### Statistical analyses

To test for variance-homogeneity and normal distribution of data a Shapiro-Wilk test as well as a Levene's test were used. Since development time was not normally distributed, differences between males and females were analysed with a Mann Whitney-*U* test.

The behavioural traits were analysed according to [3,17]. Kendall's W coefficient of concordance was calculated to analyse if larvae (L2 or L3) or adults (A1, A2 or A3), respectively, were similarly ranked across all tested behavioural traits [47]. To investigate the relation between behavioural traits within one life stage, an agglomerative cluster analysis using *agnes* function with Ward's clustering method within the R-package *irr* was conducted [48]. The likely number of behavioural dimensions was identified by a Silhouette plot [49] with the R-package *cluster*, but was not further formally tested. Kendall's W coefficient of concordance was analysed [47] both to determine the consistency across the behavioural traits measured in three contexts for larvae and four for adults within a test series and to test for consistency of each behavioural trait over time between the different life stages. To test for the consistency over time between the larval and the adult stage only the behavioural traits which were tested for both stages were used. Consistency over time was computed for each behavioural trait separately as well as for each behavioural dimension by using a composite variable, adding the ranks of each individual over all behavioural traits included in this dimension per life stage. All behavioural traits were ascendingly ranked, except the behavioural traits *dark-light* and *thanatosis 1 *which were descendingly ranked. In this way, high ranks imply bolder or more active behaviour, respectively. Because of multiple testing, the p-values within a set of tests were adjusted by using the false discovery rate [50].

To test for changes within behavioural traits over lifetime, the *amount of movements* was analysed as typical activity trait using a one way repeated measures ANOVA, followed by pairwise multiple comparisons (Holm-Sidak method), since data were normally distributed. To compare the duration of *thanatosis 1 *as typical boldness trait between the life stages we divided the individuals in two categories, those responding within 0-10 s (fast responders and thus considered as bold) and those responding within 11-300 s (slow responders and thus considered as shy). The proportion of individuals belonging to these two categories within each test phase was compared between the larval stages and between the larval and the adult stages using Fisher's exact tests, followed by a correction for false discovery rate.

To test for differences in adult behavioural dimensions between sexes and depending on age, a two way repeated measures ANOVA (sex and age as factors) followed by pairwise multiple comparisons (Holm-Sidak method) was used based on the composite variables.

To test for relationships between life-history and behavioural traits we used a Spearman's rank correlation analysis correlating the larval development time and the composite variables of the activity and the boldness dimension of L2 and L3 larvae, and A1 adults, respectively. The statistical tests were conducted by using the program R (3.0.3), STATISTICA 10 (Statsoft, Germany), and SigmaPlot 11.0 (Systat Software, USA).

## Declarations

We acknowledge financial support for this publication by the German Research Foundation (FOR 1232) and the Open Access Publication Fund of Bielefeld and Muenster University.
